# Propylene glycol neurotoxicity due to sodium citrate therapy in an infant with renal tubular acidosis 

**DOI:** 10.5414/CNCS109984

**Published:** 2020-05-08

**Authors:** Mahjabeen Khan, Ara Vartanyan, Anthony Scalzo, Sarah Riley, Jeanine Cain, Joseph Maliakkal

**Affiliations:** SSM Health Cardinal Glennon Children’s Hospital, Saint Louis University School of Medicine, Saint Louis, MO, USA

**Keywords:** propylene glycol, toxicity, renal tubular acidosis, sodium citrate

## Abstract

Sodium citrate in its liquid formulation is commonly used as therapy for renal tubular acidosis in pediatric patients. Convenient dosing and administration is important to ensure long-term medication adherence and normal growth in the chronic forms of this condition. Liquid sodium citrate formulations contain propylene glycol, a commonly used excipient, which can be toxic at high doses. Propylene glycol toxicity due to medication excipients has been reported in the literature, including many cases secondary to sustained exposure to intravenous anti-epileptics, however toxicity associated with oral sodium citrate therapy has not been described. We report the first case of propylene glycol neurotoxicity in a 6-week-old infant with renal tubular acidosis treated with sodium citrate. Clinical suspicion of risk for medication-related toxicity and awareness of propylene glycol content in sodium citrate led to timely diagnosis and management. Awareness of increased risk of toxicity in pediatric patients due to high sodium citrate requirement and low propylene glycol metabolism capacity is important for optimal care for pediatric patients with renal tubular acidosis.

## Introduction 

Propylene glycol (PG) is a commonly used excipient in many medications. Toxicity due to PG has been described in the literature, mainly with prolonged iatrogenic exposure to anti-epileptics [1, 2, 3]. Other drugs reported to be associated with PG toxicity are silver sulfadiazine, etomidate, and multivitamins [4]. We report a unique case of PG toxicity manifesting with altered mental status in a 6-week-old infant with renal tubular acidosis (RTA) treated with citrate supplements containing PG. Common use of citrate salts in the management of renal tubular acidosis requires that providers consider the risk of PG toxicity, especially in the pediatric population. 

## Case presentation 

**Figure 1. Figure1:**
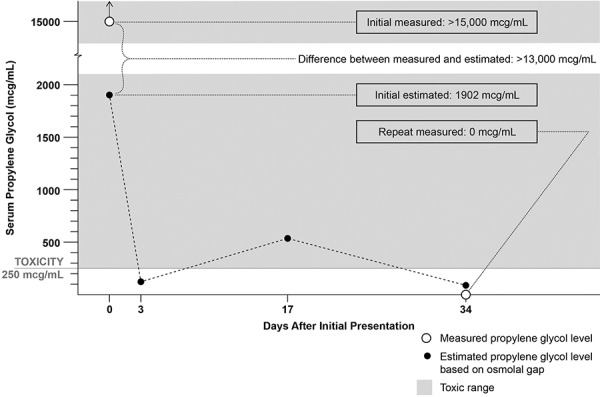
Graph depicting the changes in estimated and measured serum propylene glycol levels in relation to days after initial presentation.

A full-term 4-week old, African American male with an admission weight of 3,535 g presented to the emergency department with fever of 38.3 °C and nasal congestion. The patient’s mother reported no diarrhea, emesis, or cough and a recent diagnosis of thrush with initiation of nystatin therapy 2 days prior to presentation. Family history was significant for the mother, maternal uncle, and additional maternal relatives with renal tubular acidosis treated with bicarbonate supplements; however, the specific cause of RTA was never diagnosed. On admission, physical examination revealed a small appearing neonate with decreased subcutaneous fat in mild respiratory distress. An evaluation for sepsis was performed. Chemistry studies revealed serum bicarbonate of 5 mmol/L, serum sodium of 132 mmol/L, serum potassium of 3.4 mmol/L, serum chloride 114 mmol/L, serum creatinine of 0.49 mg/dL, blood urea nitrogen 19.3 mg/dL, serum phosphorus 5 mg/dL, and serum magnesium of 2.5 mg/dL. Venous blood gas revealed pH 7.16 and pCO_2_ of 19 mmHg. Urinalysis was notable for urine pH 6.0 and negative for glucose. These results were consistent with non-anion gap metabolic acidosis. Fractional excretion of bicarbonate was appropriate at 1%. The absence of diarrhea, lack of evidence of proximal tubular dysfunction (normal serum phosphorus, appropriate fractional excretion of bicarbonate, and absence of glucosuria), presence of low serum potassium, and the finding of inappropriately high urine pH led to clinical diagnosis of distal renal tubular acidosis. Intravenous sodium acetate was initiated with improvement in serum bicarbonate levels. Renal ultrasound showed normal kidneys with no evidence of nephrocalcinosis. The patient was discharged on oral sodium citrate 8.8 mEq/kg/day (10 mL 3 times daily) and potassium citrate supplementation 4.4 mEq/kg/day (2.5 mL 3 times daily) with plan for close outpatient follow-up. The bicarbonate supplementation formulation included sodium citrate solution 500 mg/334 mg per 5 mL (1 mL = 1 mEq of sodium ion and 1 mEq bicarbonate equivalent of citrate) and the potassium citrate and citric acid solution 1,100 mg/334 mg per 5 mL (1 mL = 2 mEq potassium ion and 2 mEq bicarbonate equivalent of citrate). Both solutions contained 2% v/v PG.[Fig Figure1]

The evening after hospital discharge, the patient’s mother brought him back to our emergency room for concerns of lethargy. His mother stated that he appeared more tired and did not wake to feed. She noted that he was staring and blinking with his hand in his mouth, but denied noticing any shaking, tremor, emesis, skin color changes, or breathing issues. In the emergency room, the patient had a witnessed episode of staring without changes in vital signs, shaking, jerking, or emesis. CBC, CMP, and phosphorous levels were obtained and unremarkable. The patient was admitted for observation and evaluation of staring spells. During hospitalization, he returned to his baseline level of activity without initiation of anti-epileptic therapy and no additional staring spells were noted. 

Upon medication review by a clinical pharmacist, concerns arose for possible excess exposure to PG. The PG content of sodium citrate and potassium citrate supplementation was verified with the manufacturer and estimated at 222 mg/kg/day, higher than the WHO limit for PG in food additives at 25 mg/kg/day. This high dose of citrate therapy was likely being administered for ~ 6 days prior to the onset of his symptoms. Review of medications for additional exposure to PG revealed that nystatin, swabbed to each side of mouth for treatment of thrush beginning 4 days prior to admission, also contained PG. Oral citrate supplementation and nystatin were stopped, and intravenous fluid with acetate was started. Toxicology service was consulted and PG levels were found to be elevated at > 15,000 µg/mL, a level consistent with PG toxicity and expected to cause altered mental status. Measured serum osmolality on admission was elevated at 306 mOsm/kg, and the calculated osmolal gap was 25 mOsm/kg. Serum osmolality 1 week later was normal at 283 mOsm/kg. The patient was then transitioned from intravenous sodium acetate to oral sodium bicarbonate supplementation (medication with no PG), and the dose was titrated to achieve a goal venous blood gas pH 7.35 – 7.45. The patient was transitioned back to oral sodium citrate supplementation at 1.3 mEq/kg/day. Two weeks following discharge, PG level was undetectable, and venous blood gas showed pH was in goal range at 7.36. 

## Discussion 

PG is a commonly used excipient in many medications, including intravenous, oral, and topical formulations. The United States Food and Drug Administration (FDA) ruled PG as “generally recognized as safe” [5]; however, adverse effects from PG have been reported. Clinical findings include seizures, cardiotoxicity, CNS toxicity, hyperosmolarity, and lactic acidosis. Several drugs are implicated in PG toxicity, including anti-epileptics, sedatives, topical silver sulfadiazine, and multivitamins used in total parenteral nutrition [4]. To our knowledge, this is the first reported case of PG neurotoxicity associated with sodium citrate therapy. 

PG toxicity can cause lactic acidosis as it is metabolized by alcohol dehydrogenase to form lactate. Lactate undergoes conversion to pyruvate and enters the citric acid cycle, and ~ 12% undergoes renal elimination [3]. Lactic acidosis resulting from prolonged infusion of anti-epileptics in critically ill patients with likely some degree of renal insufficiency has been described in case reports [1, 2, 3]. Unlike anti-epileptics, sodium citrate formulations containing PG may mask lactic acidosis due to the alkalinizing effect of citrate. 

Correction of metabolic acidosis from RTA is important in children to ensure normal growth, reduce the risk of osteopenia and minimize nephrocalcinosis. Weight-adjusted doses of citrate salts for treatment of distal RTA are lower in adults (1 – 4 mEq/kg/day) [6] compared to infants in the first year of life (up to 10 mEq/kg/day) [7]. This higher alkali therapy requirement is due to larger urinary bicarbonate loss in children and the increased acid generated from the growing skeleton, after correction for body surface area. Higher doses of citrate supplementation expose children to an increased risk of PG toxicity. Pharmacokinetics of PG in children also put them at a higher risk of toxicity because the mean half-life of PG in children is much longer than in adults (19.3 hours versus up to 3.3 hours) [8, 9]. Decreased renal clearance with increased dosing of PG in children has also been reported [10]. 

Delays in definitive diagnosis of the rare phenomenon of PG toxicity are at risk for further postponement by the requirement of specimen submission to specialized laboratories [11]. Use of osmolal gap as a screening tool can expedite the diagnosis of PG toxicity. PG is osmotically active, and high serum levels lead to increased serum osmolality. Using this principle, an elevated osmolal gap (difference between calculated and measured osmolality > 10 mOsm/kg) can estimate the PG serum level in situations where direct measurement of serum concentration is not immediately available [12]. The only pediatric formula is based on a case report of an 8-month-old with PG toxicity from topical sulfa ointment use. Fligner et al. [13] used linear regression to describe the relationship between the osmolar gap and the measured propylene glycol levels. The following regression equation was reported: 

[PG concentration (mg/dL)] = 84.6 + 7.8 [osmolal gap (mOsm/kg)] 

Fligner et al. [13] concluded that regression equation showed that PG concentration contributes to the osmolal gap in a manner consistent with the molecular weight of propylene glycol (76.095 Da) and expectation that 1 mmol/L contributes 1 mOsm/kg to the serum osmolality. The following is the empiric equation based on the observations of Fligner et al. [13]: 

PG concentration (µg/mL) = [osmolal gap (mOsm/kg)] × 76.095 (kg×µg/mOsm×mL) 

However, in the case of our patient, the measured PG concentration (> 15,000 µg/mL) was much higher than the concentration estimated by the osmolal gap (1,902 µg/mL). We speculate that the difference between the measured PG concentration and the concentration estimated by the osmolal gap was high due to the extraordinarily high measured PG concentration. In fact, in order for the measured level of > 15,000 µg/mL to be estimated correctly, the osmolal gap would have been 197 mOsm/kg, and the measured serum osmolality would have been 478 mOsm/kg (normal serum osmolality ~ 290 – 300 mOsm/kg). Given that these measurements may have been beyond the ability of the lab equipment to measure reliably, we speculate that this contributed to the large difference between the measured and estimated levels. 

There is no formal recommendation in the United States for a limit for PG intake from medications. Our patient was receiving 222 mg/kg/day of PG. WHO recommends a safe limit for PG in food additives at 25 mg/kg/day. European Medicine Agency (EMA) proposes a tolerance limit of 1 mg/kg/day for neonates and 50 mg/kg/day for up to 4 years of age [14]. Evidence is lacking for safe limits for PG exposure in the pediatric population and conjecture from adult data is inadequate based on cases of toxicity at doses lower than extrapolated thresholds [15, 16]. In our review of the manufacturer-reported content of sodium and potassium citrate products, all preparations contain PG. Out of the six commercial preparations we looked at, only three reported the actual content of PG (2% v/v PG). 

In summary, we report the first case of PG toxicity due to citrate supplementation. High citrate dosing combined with low excipient clearance contributed to increased susceptibility of our pediatric patient to PG toxicity. Awareness of PG content and clinical suspicion for presenting findings led to successful diagnosis and management. A 1.2 mL/Kg/day of sodium or potassium citrate would exceed the limit of 25 mg/kg/day of PG (assuming 2% v/v PG) and could lead to toxicity. Serum osmolality helped as a screening tool, and the diagnosis was confirmed by serum PG measurement. Labeling of PG content in all medications would increase awareness of exposure. Additional studies are needed to characterize metabolism and toxicity of PG in pediatric patients. 

## Funding 

There was no support/funding for this report. 

## Conflict of interest 

There were no potential conflicts of interest noted.
